# Supplementary material for: N-Doped Graphene-like Film/Silicon Structures as Micro-Capacitor Electrodes
Source: Materials (Basel). 2023 May 26;16(11):4007. doi: 10.3390/ma16114007 (PMC10254910; doi:10.3390/ma16114007)

Supplementary Materials

N-Doped Graphene-like Film/Silicon Structures as Micro-Capacitor Electrodes

Daria M. Sedlovets

Institute of Microelectronics Technology and High-purity Materials, Russian Academy of Science, Chernogolovka, Moscow District, 6 Academician Ossipyan Str., 142432 Chernogolovka, Russian; sedlovets@iptm.ru; Tel.: +7-4965244190

Section S1. Graphene-like film synthesis

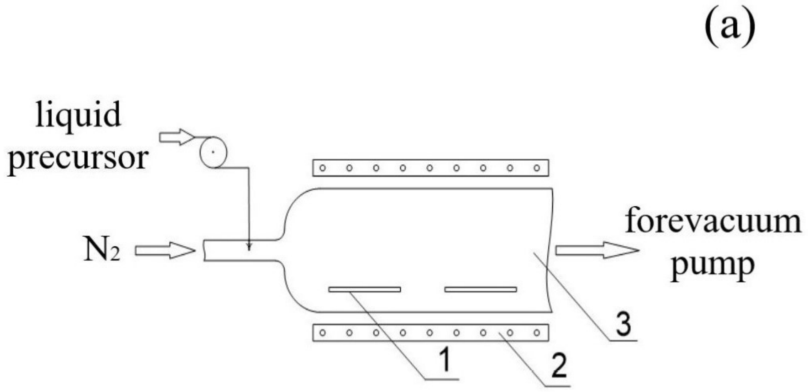

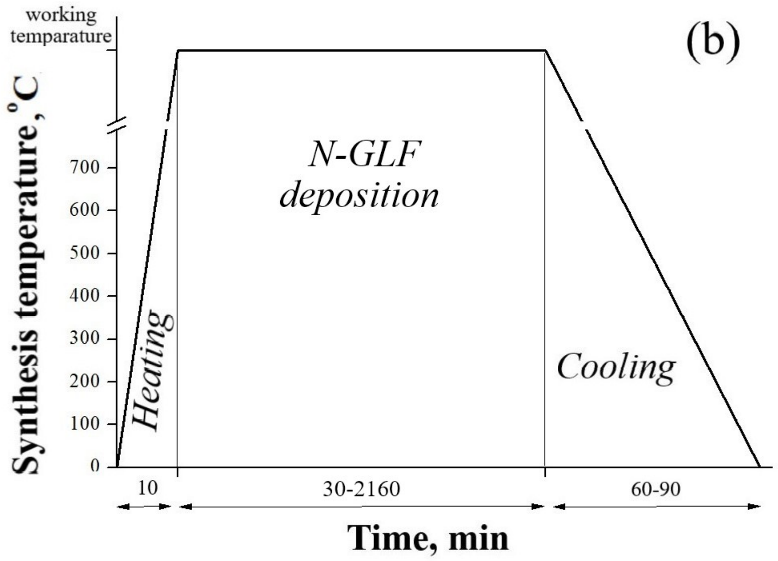

Figure S1. Schematic of the lab-made setup used to grow GLFs from acetonitrile or ethanol vapor: 1 – substrates, 2 – external electric furnace, 3 – quartz reactor (a). The synthesis diagram (b).

Table S1. Sample specification.

**Material**	**Precursor**	**In-text description**	**Temp., °C**	**Time, h**	**ρ, kΩ/□**	**Figures**
N-GLF	CH_3CN	thin	800	26	~ 10	1a, 1c
N-GLF	CH_3CN	thin	900	17	~ 10	1a, 1c
N-GLF	CH_3CN	thin	1000	0,5	~ 10	1a, 1c, 3b
N-GLF	CH_3CN	thick	800	54	~ 100	1b, 1d
N-GLF	CH_3CN	thick	900	26	~ 100	1b, 1d, 2a, 3a
N-GLF	CH_3CN	thick	1000	2	~ 100	1b, 1d
N-GLF	CH_3CN	prolonged	900	35	~ 2-3	2b, 3a
GLF	C_2H_5OH	non-doped	1000	0,5	~ 100	3b

Section S2. CV curves

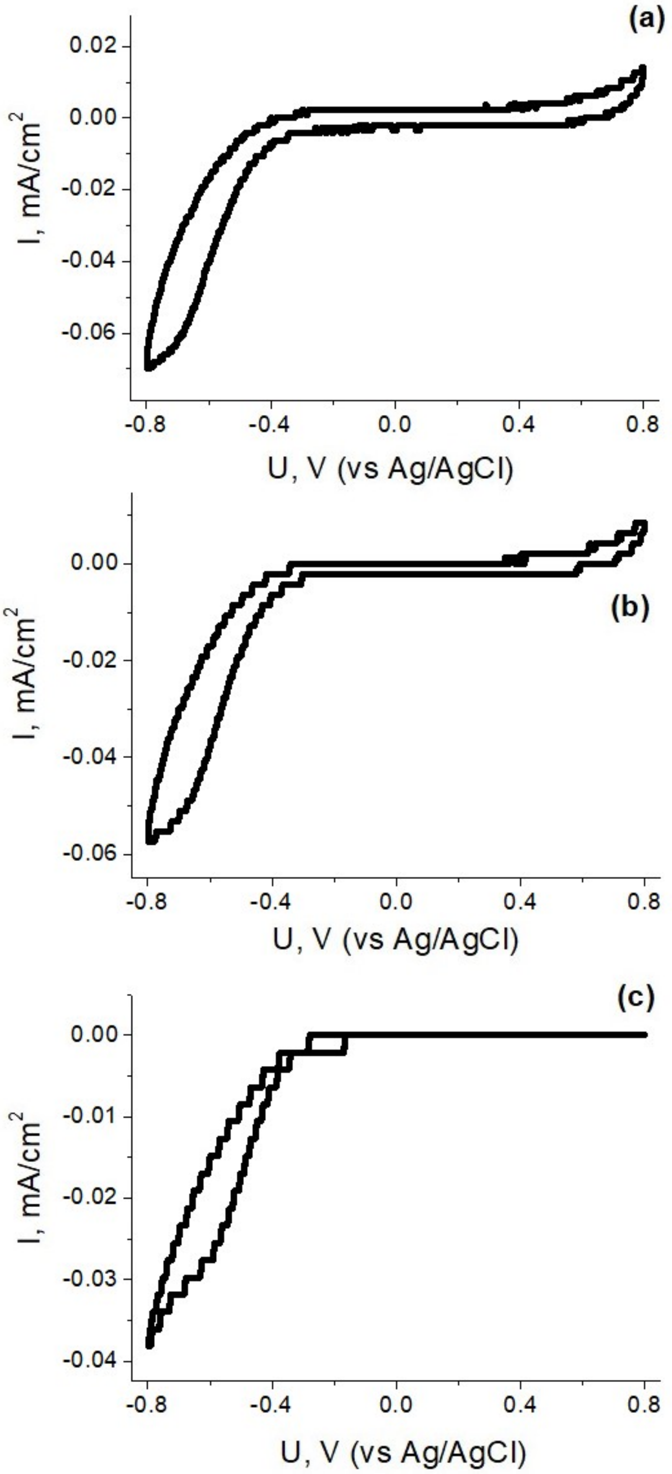

Figure S2-0. CV curves for thick N-GLFs with sheet resistances of ~10 kOhm/square, synthesized at 1000 °C. Measurements performed before soaking in electrolyte at various scan rates: 100 mV/s (a), 50 mV/s (b) and 5 mV/s (c).

CV curves for N-GLFs at various scan rates: 100 mV/s (red), 50 mV/s (blue), 20 mV/s (green), 10 mV/s (magenta) and 5 mV/s (black).

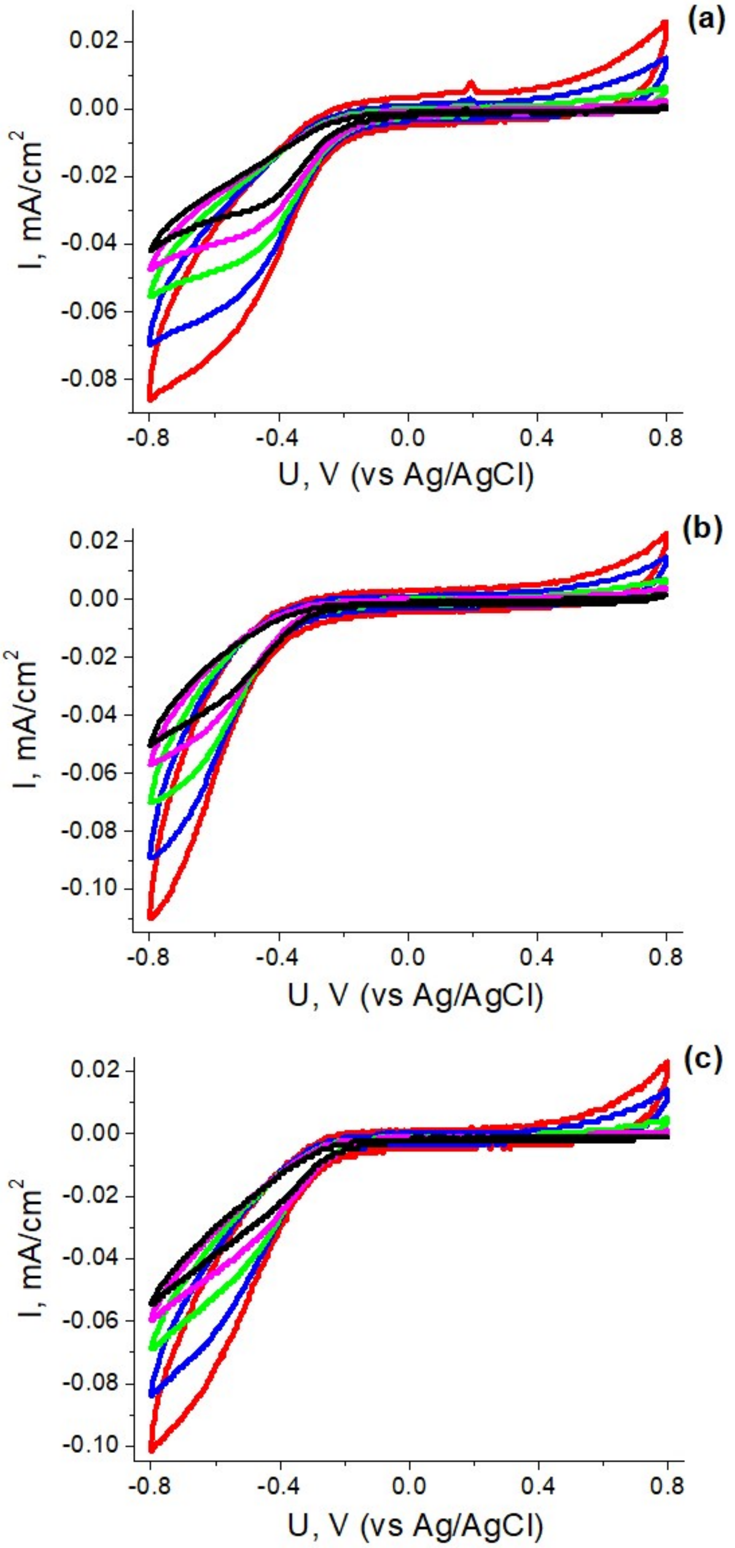

Figure S2-1. CV curves for thin N-GLFs with sheet resistances of about 100 kOhm/square. Synthesis temperature: 800 °C (a), 900 °C (b), 1000 °C (c).

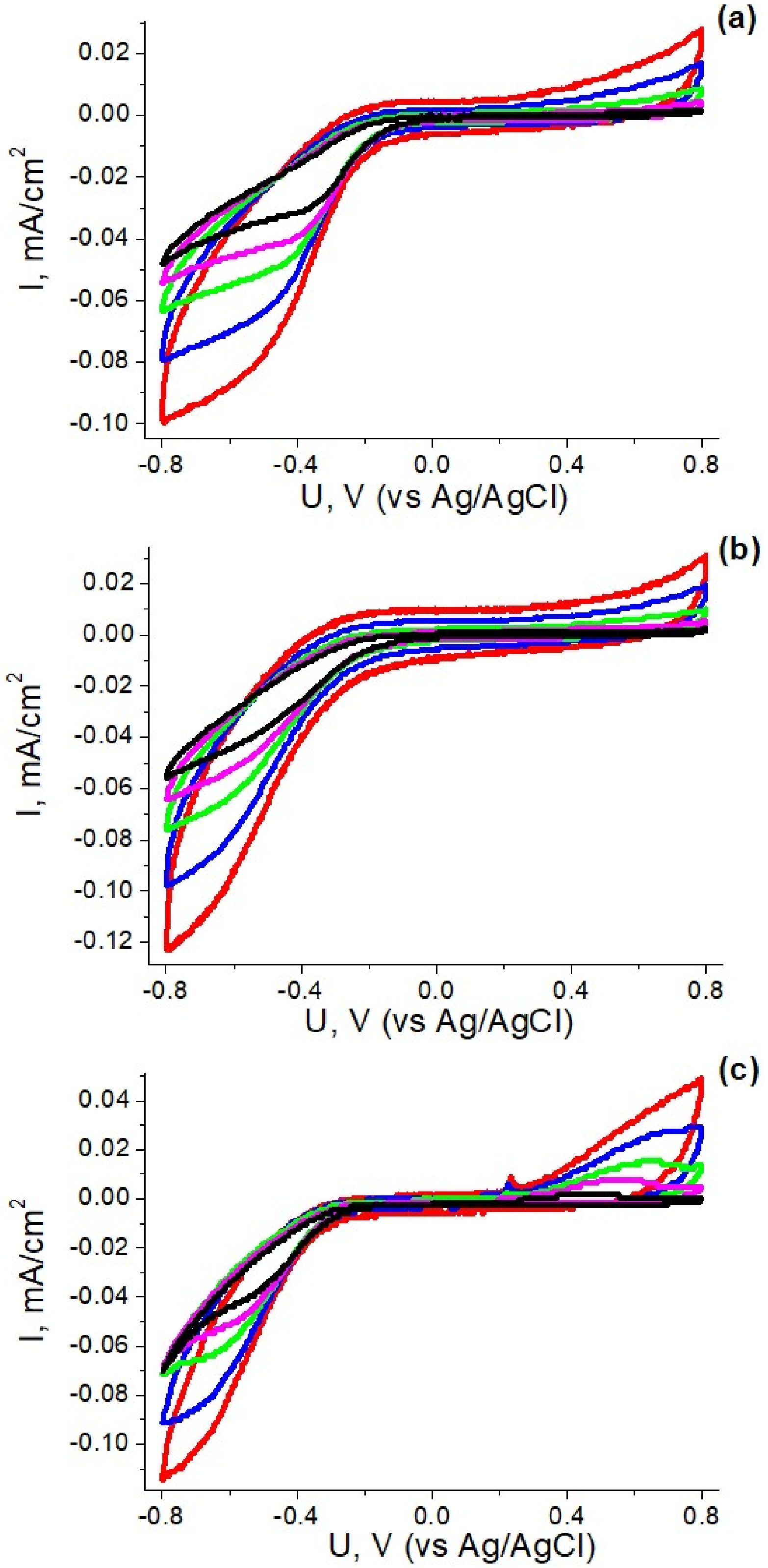

Figure S2-2. CV curves for thick N-GLFs with sheet resistances of about 10 kOhm/square. Synthesis temperature: 800 °C (a), 900 °C (b), 1000 °C (c).

Section S3. Raman characterization

It has recently been reported (https://doi.org/10.1016/j.materresbull.2022.111943) that the nanocrystalline structure of N-GLFs causes deviations of their spectra from the "classical" spectrum of high-quality graphene, namely: the appearance of the “defect” peaks D (band originating from sp^2 lattice disturbance at ~1350 cm^{-1}); broadening and weakening of G (at ~1600 cm^{-1}) and 2D (at ~2750 cm^{-1}) main peaks. Micro-inhomogeneity of the films can complicate the analysis of their Raman spectra. No visible discrepancy between the spectra was detected and subsequent quantitative analysis was performed: the baseline subtraction and spectra fitting were done followed by peak areas extraction.

The ratios of D and G band intensities were calculated to estimate the structural perfection. I_D/I_G ratio is inversely proportional to an average crystallite size. As seen from Figure S3, the I_D/I_G ratio was is weakly, but statistically significant anticorrelated with temperature. This means that higher temperatures result in higher crystallinity of the films.

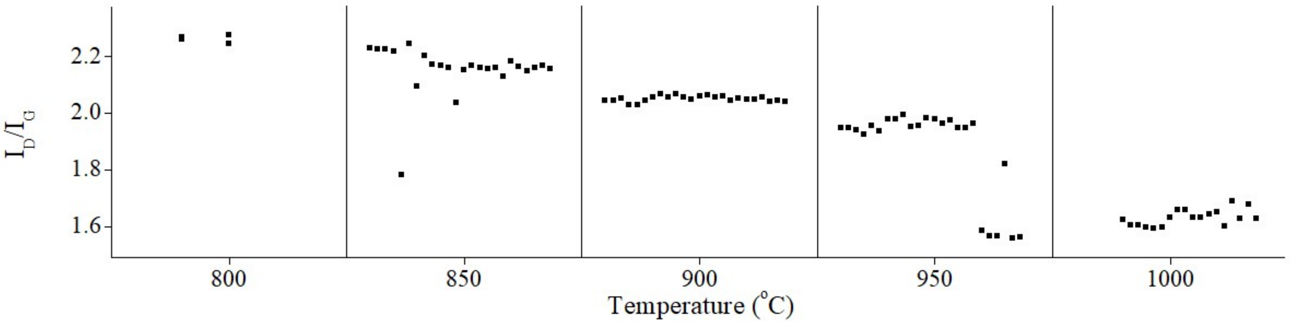

Figure S3. Dependence of I_D/I_G values on the synthesis temperature for N-GLFs on SiO_2.

Section S4. XPS spectra

Processing of individual line N1s has been performed to exactly determine the local structure of the nitrogen inclusions. There are three major types of N heteroatom bonding configurations within the carbon lattice: sp^3 pyrrolic (1), sp^2 pyridinic (2) edge states and quaternary/graphitic (3) N attributed to atoms substituting carbon in the benzene ring. The N1s bands were convoluted on three components with the following positions: 398.7 ± 0.2 eV, 400 ± 0.2 eV and 401.2 ± 0.2 eV which refer to pyrrolic, pyridinic and graphitic N, respectively. Convoluted spectra are shown in Figure S4. The data on the relative content of each nitrogen configuration in N-GLFs deposited at different temperatures are provided in main text (Table 1).

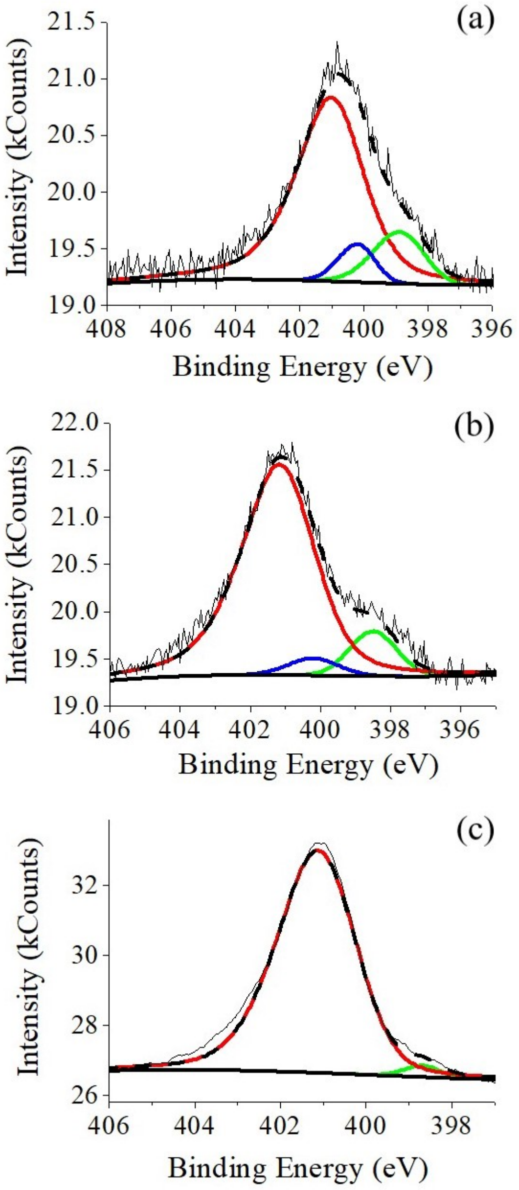

Figure S4. Convoluted N1s spectra of N-GLFs deposited at 800 °C (a), 900 °C (b), 1000 °C (c). Red line: graphitic N; blue line: pyridinic N; green line: pyrrolic N.

Section S5. GCD curve

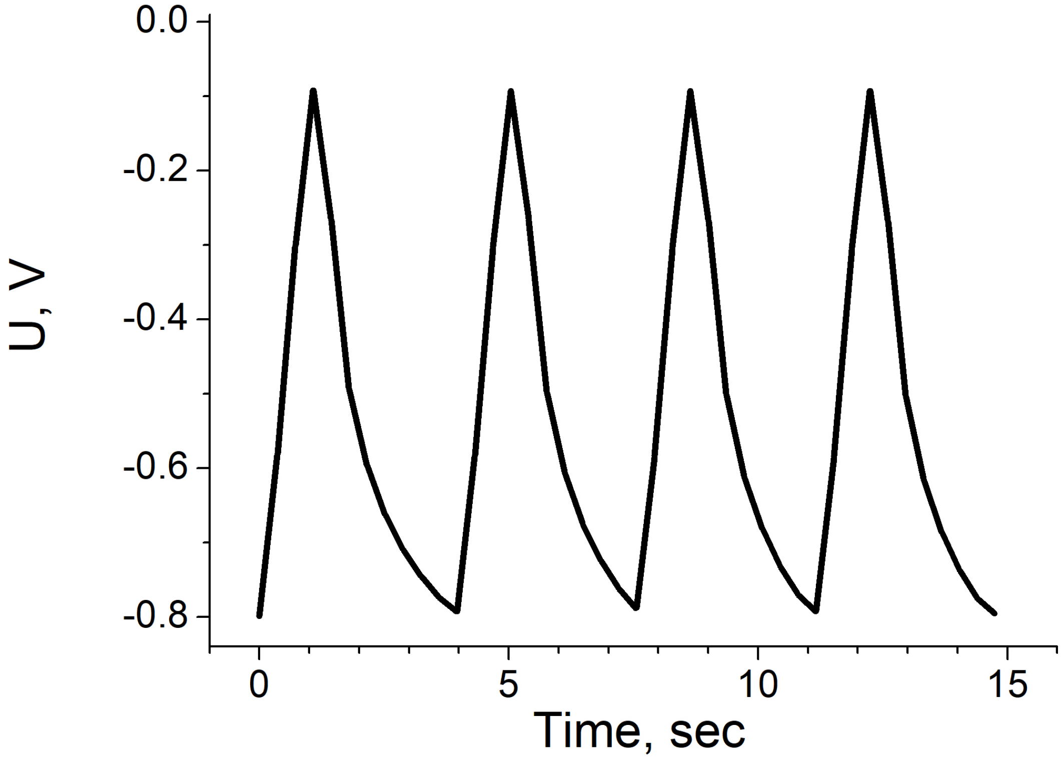

Figure S5. GCD curve for thick N-GLFs synthesized at 900 °C, measured after 5000 cycles at 0.09 mA/cm^2 .

When measured in galvanostatic mode, specific capacitance was calculated from GCD function as follows:

$$C = \frac{I_c \cdot \Delta t}{\Delta V \cdot \mathrm{A}}$$

where C is the area-normalized capacitance in F/cm^2,

I_c is the charging current in A,

Δt is the discharge time in s,

ΔV is the scanned potential window in V (excluding IR drop),

A is the surface area which was exposed to electrolyte, in cm^2.

According to Figure S5, the capacitance was found to be about 200 μF/cm^2. This correlates very well with the value obtained from the CV curve, considering the rate of voltage change of equal to about 200 mV/s.

Section S6. AFM images

The thickness was estimated as the height difference at the film-substrate interface from cross-section data obtained with Integra AFM.

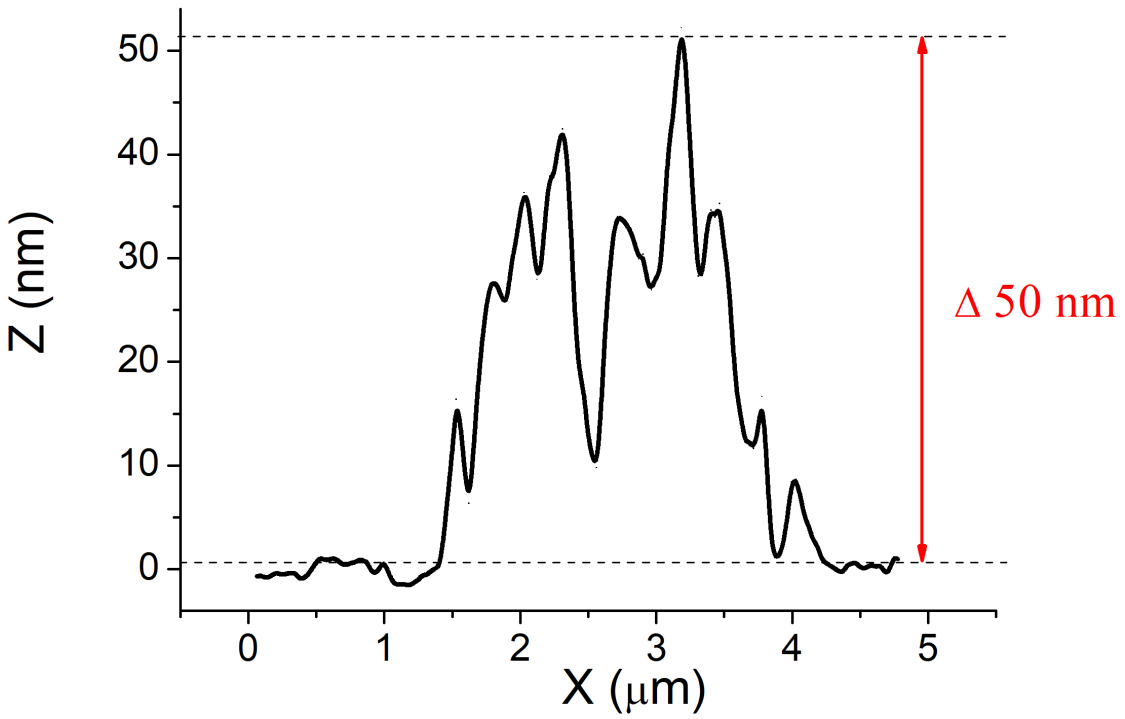

Supplement: Supplementary file 1 [file materials-16-04007-s001.zip › materials-2210252-supplementary.pdf]
